# Effects of Sitagliptin on Lipid Profiles in Patients With Type 2 Diabetes Mellitus: A Meta-analysis of Randomized Clinical Trials

**DOI:** 10.1097/MD.0000000000002386

**Published:** 2016-01-15

**Authors:** Minhua Fan, Yuelan Li, Shihong Zhang

**Affiliations:** From the Department of Nursing, Liaocheng People's Hospital, Liaocheng, Shandong, PR China.

## Abstract

Sitagliptin has been reported to improve lipid profiles, but findings from these studies are conflicting. We conducted this meta-analysis to evaluate the effects of sitagliptin on serum lipids in patients with type 2 diabetes mellitus.

We made a comprehensive literature search in PubMed, EMBASE, Cochrane Library, China National Knowledge Infrastructure, Wanfang, and VIP database until June 2015. Eligible studies were randomized clinical trials (RCTs) that investigated the effect of sitagliptin on serum triglycerides (TGs), total cholesterol (TC), low-density lipoprotein cholesterol (LDL-C), or high-density lipoprotein cholesterol (HDL-C).

Eleven RCTs with 2338 patients were identified. Compared with controls, sitagliptin alone or in combination significantly improved serum TG (weighted mean difference [WMD] −0.24 mmol/L; 95% confidence interval [CI] −0.40 to −0.09; *P* = 0.002) and HDL-C (WMD 0.05 mmol/L; 95% CI 0.02–0.07; *P* < 0.001).However, no statistical significances were observed in LDL-C (WMD −0.07 mmol/L; 95% CI −0.22 to 0.08; *P* = 0.337) and TC (WMD −0.14; 95% CI −0.33 to 0.06; *P* = 0.177). Subgroup analyses revealed that sitagliptin alone achieved greater improvement in serum TG, TC, and HDL-C levels.

These findings suggested that sitagliptin alone or in combination significantly improved serum TG and HDL-C levels in patients with type 2 diabetes mellitus.

## INTRODUCTION

Diabetes mellitus is one of the most common chronic diseases worldwide. Diabetes mellitus is a well-known risk factor for cardiovascular disease. Dyslipidemia is an essential determinant of cardiovascular risk in type 2 diabetes.^1^ Patients with type 2 diabetes are more likely to be dyslipidemic than the general population.^2^ Lipid abnormalities in patients with type 2 diabetes are characterized as high serum triglyceride (TG), low-density lipoprotein cholesterol (LDL-C), as well as total cholesterol (TC),and reduced serum high-density lipoprotein cholesterol (HDL-C) concentration.^3^ The coexistence of diabetes and lipid abnormalities can further increase the risk of cardiovascular disease. Antihyperglycemic agents had positive effects on the dyslipidemia and may contribute to reduce the excessive risks of cardiovascular events in a person with diabetes.

Dipeptidyl peptidase-4 (DPP-4) inhibitors are a class of oral antihyperglycemic agents for treatment of type 2 diabetes.^4^ Treatment with DPP-4 inhibitor alone or in combination with other antihyperglycemic agents can improve the success in achieving and/or maintaining glycemic control in patients with type 2 diabetes. In addition to an improvement in glycemic control, DPP-4 inhibitor appeared to have a beneficial effect on dyslipidemia. On the basis of previous clinical evidence, a well-designed meta-analysis^5^ confirmed that DPP-4 inhibitors as a class drug had beneficial effects on TC and TG and different DPP-4 inhibitors had differential effects on the lipid profile. Sitagliptin is the first selective DPP-4 inhibitor clinically used in 2006.^6^ Sitagliptin had pleiotropic effects in the treatment of patients with type 2 diabetes, including improved lipid parameters,^7^ but findings are inconsistent across studies. Therefore, whether sitagliptin could be effective in improving lipid abnormalities is controversial.

Here, we conducted a meta-analysis of the available randomized controlled trials (RCTs) to determine the positive benefits of sitagliptin on lipid abnormalities in patients with type 2 diabetes.

## METHODS

### Literature Search

This meta-analysis was performed in accordance with the Preferred Reporting Items for Systematic Reviews and Meta-analyses (PRISMA) statement.^8^ We searched PubMed, EMBASE, Cochrane Library, China National Knowledge Infrastructure, Wanfang, and VIP database from inception until July 2015. We used the following search terms with various combinations: sitagliptin [Medical Subject Headings (MeSH)] OR DPP-4 inhibitor [Mesh] OR dipeptidyl peptidase-4[Mesh] AND diabetes mellitus [Mesh] OR type 2 diabetes [Mesh] AND (randomized [Free Item] OR randomized [Free Item] OR randomization [Free Item]. In addition, we also manually searched reference lists from articles to identify additional eligible papers.

### Study Selection

Studies were included according to the following criteria: RCTs enrolling patients with 2 diabetes; patients who received sitagliptin alone or in combination with other antihyperglycemic agents; and least reported the changes of serum TG, LDL-C, TC, or HDL-C change as an outcome measure. Studies were excluded if lipid profile indexes were not reported and the study design was review, abstracts, or duplicated publication.

### Data Extraction and Quality Assessment

Data for meta-analysis were extracted independently by two authors (M.H.F. and Y.L.L.). The following data were extracted from the included RCTs: first author's surname, country, year of publication, study design, sample size, mean age or age range of participants, regimen of sitagliptin and controls, follow-up period, and outcomes (serum TG, LDL-C, TC, or HDL-C levels). Any disagreement was solved through discussion. The quality of the included trials was assessed using the Cochrane risk bias tools (Revman 5.2; www.cochrane.org/training/cochrane-handbook). The risk of bias tools included selection bias (random sequence generation and allocation concealment), performance bias (blinding of participants and personnel), detection bias (blinding of outcome data), attrition bias (incomplete outcome data), reporting bias (selective reporting), and other sources of bias.

### Data Synthesis and Analysis

Lipid parameters were pooled as the weighted mean difference (WMD) with 95% confidence interval (CI). All the comparisons were made as sitagliptin alone or in combination with other antihyperglycemic agents versus control or other antihyperglycemic agents. Heterogeneity was assessed using the *I*^2^ statistics and Cochrane's *Q* test. On the basis of the Cochrane Handbook for Systematic Review of Interventions, *I*^2^ statistics >50% or *P* < 0.10 in Cochrane's *Q* test were defined as substantial heterogeneity.^9^ A random effect model was chosen when substantial heterogeneity was observed. Otherwise, we selected a fixed-effect model. We conducted subgroup analyses on the basis of the type of intervention (sitagliptin alone or in combination with other antihyperglycemic agents) and treatment duration (<18 weeks or ≥18 weeks). To estimate possible publication bias, we conducted both Begg rank correlation test^10^ and Egger regression test.^11^ All the analyses were performed using STATA statistical software (version 11.0; StataCorp, College Station, TX). A *P* value <0.05 was used to indicate statistical significance.

## RESULTS

### Search Results

**FIGURE 1:**
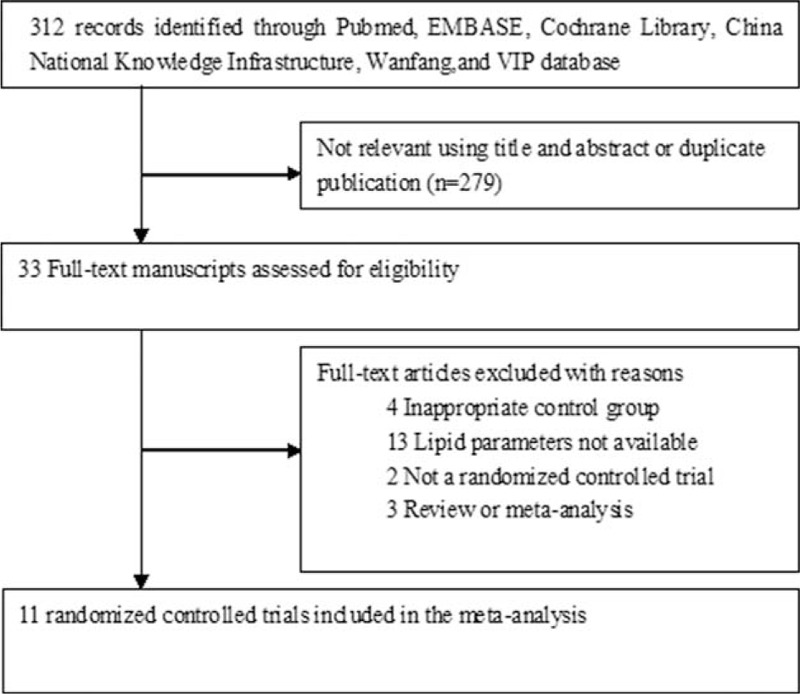
Flow diagram of trials selection process.

The initial search yielded 312 potential citations. Of these, 281 were discarded after reviewing titles and abstracts or removing duplicate publication. After reading the full-text articles, we further excluded 22 papers for various reasons. Finally, a total of 11 RCTs^12–22^ satisfied the inclusion criteria. A flow chart showing a detailed process of trial selection is provided in Figure 1.

### Characteristics of Eligible Studies

**FIGURE 2:**
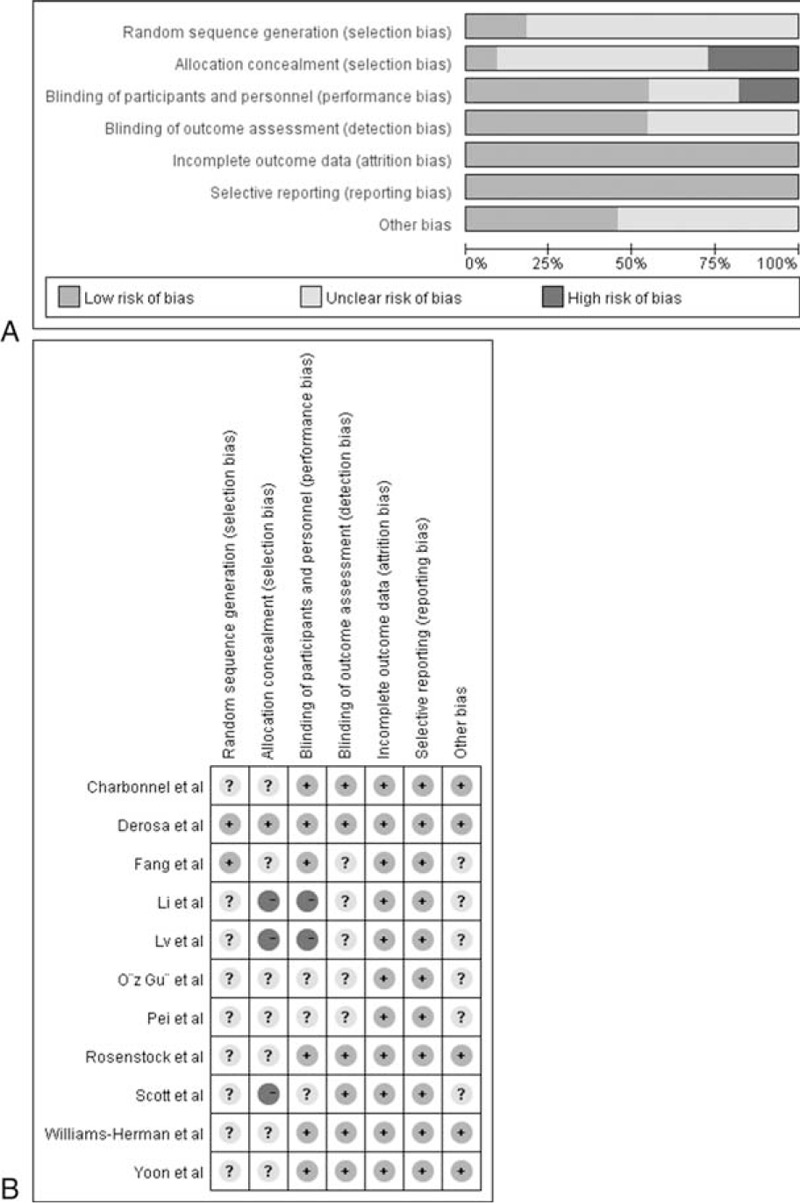
Risk of bias graph (A) and risk of bias summary (B).

The characteristics of the included RCTs are presented in Table 1 . A total of 2338 persons with diabetes were included in this meta-analysis. Of these patients, 1283 received sitagliptin alone or in combination with other antihyperglycemic agents, and 1055 assigned to controls. There was no significant difference in baseline serum lipid parameters between the 2 groups. Treatment period varied from 12 to 104 weeks. Overall, the included trials were generally classified as moderate to high quality. However, randomization and sequence concealment was not reported in most of the included studies, and the selection bias could not be excluded. The detailed quality assessment of individual trial is presented in Figure 2.

**TABLE 1:**
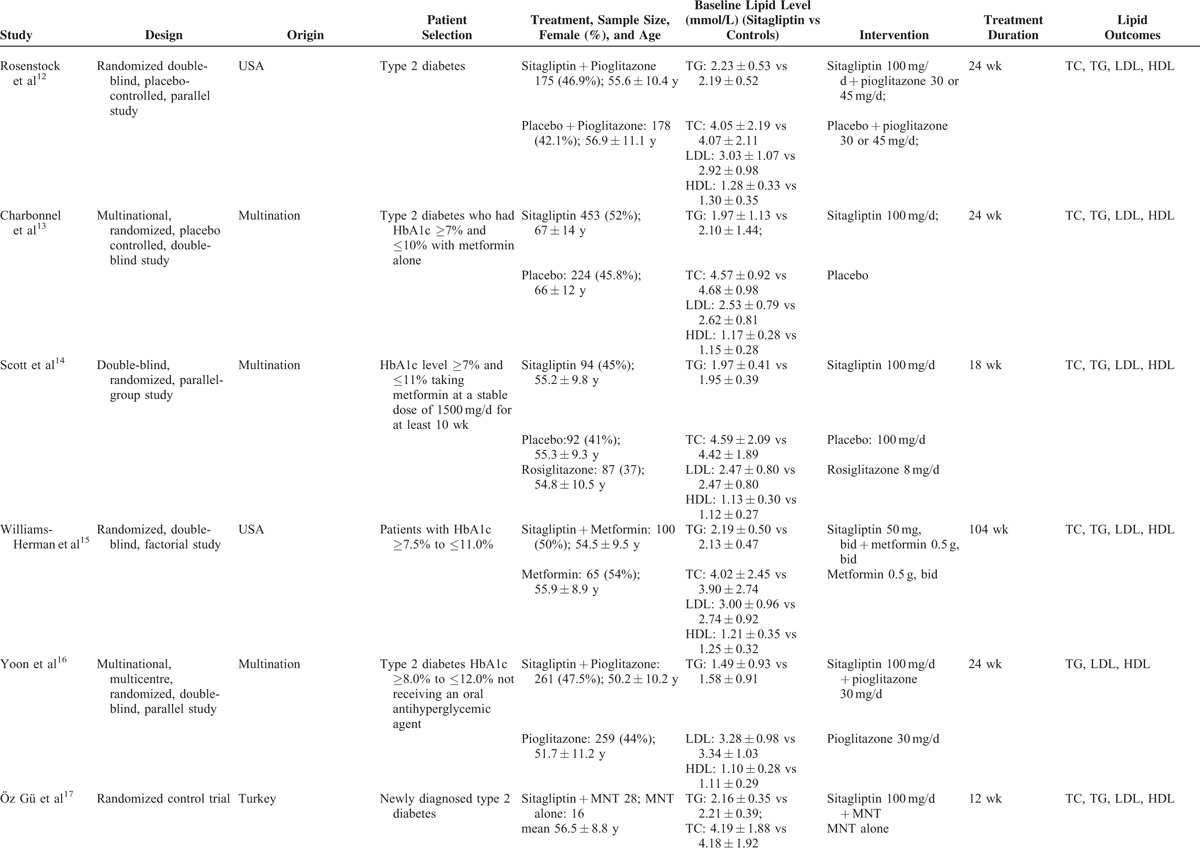
Characteristics of Clinical Trials Included in Meta-analysis

**TABLE 1 (Continued):**
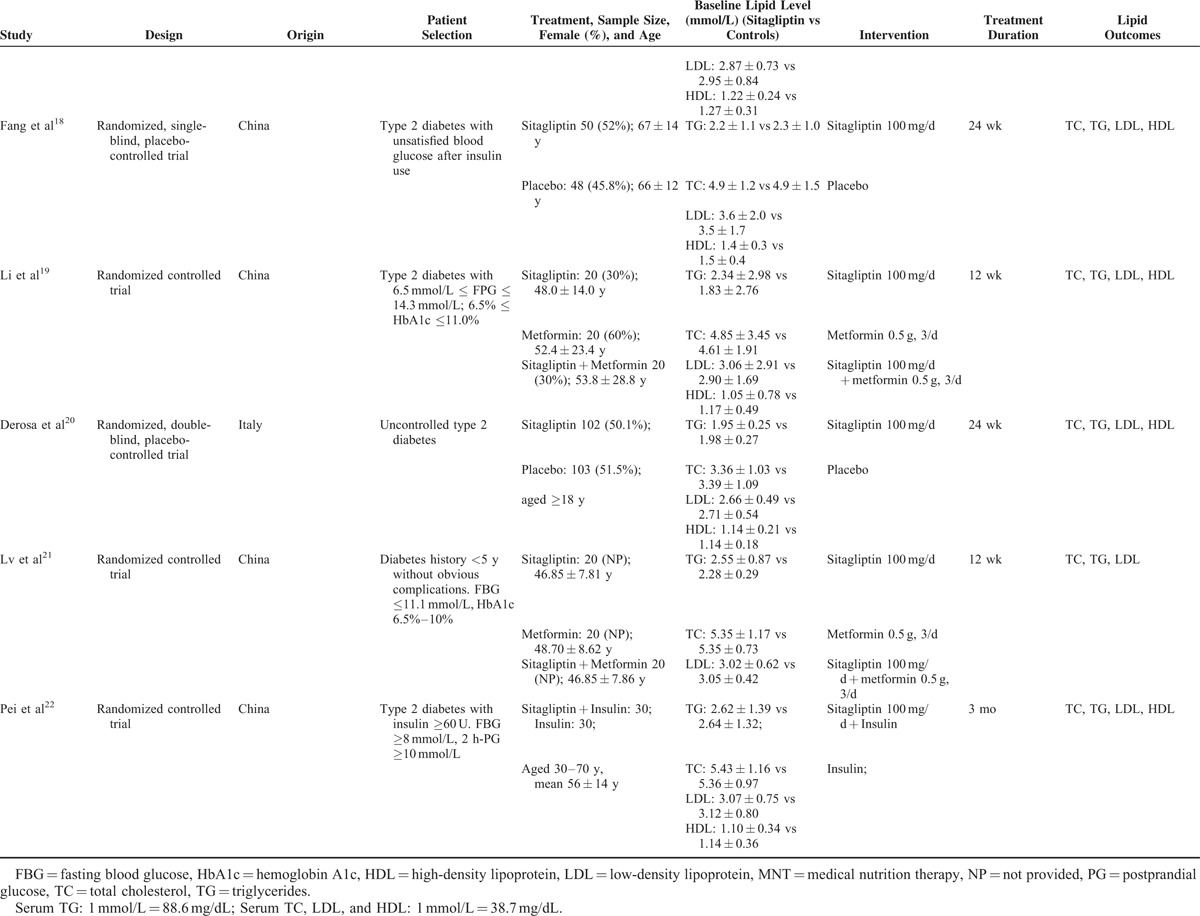
Characteristics of Clinical Trials Included in Meta-analysis

### Comparison of Serum Lipid Parameters

**FIGURE 3:**
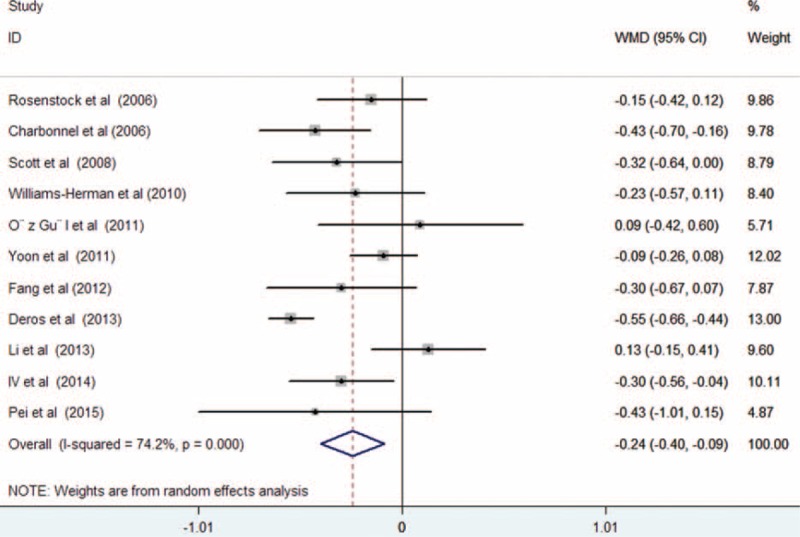
Forest plots showing weighted mean difference and 95% confidence interval for serum triglycerides levels comparing sitagliptin alone or in combination with other antihyperglycemic agents to controls in a random effects model.

**FIGURE 4:**
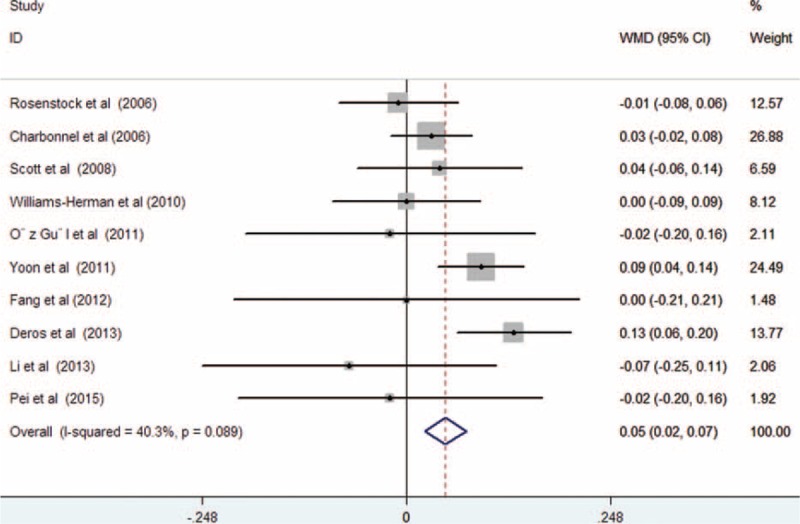
Forest plots showing weighted mean difference and 95% confidence interval for serum high-density lipoprotein cholesterol levels comparing sitagliptin alone or in combination with other antihyperglycemic agents to controls in a random effects model.

**FIGURE 5:**
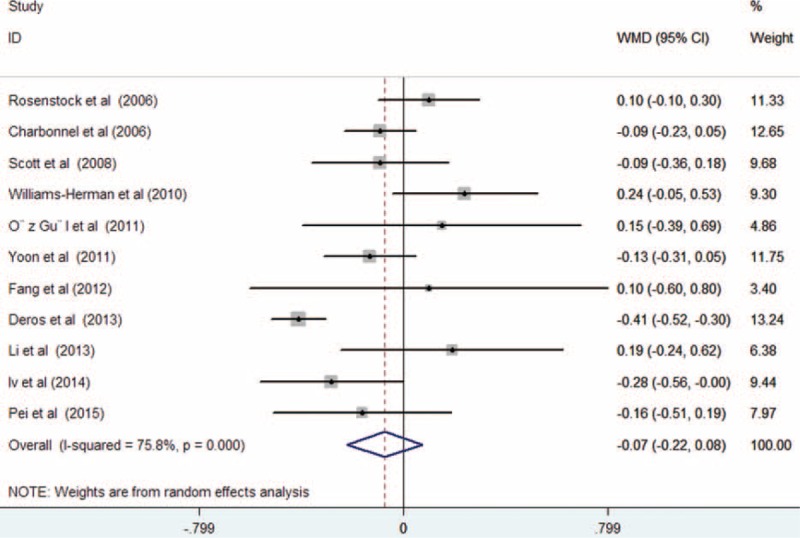
Forest plots showing weighted mean difference and 95% confidence interval for serum low-density lipoprotein cholesterol levels comparing sitagliptin alone or in combination with other antihyperglycemic agents to controls in a fixed-effect model.

**FIGURE 6:**
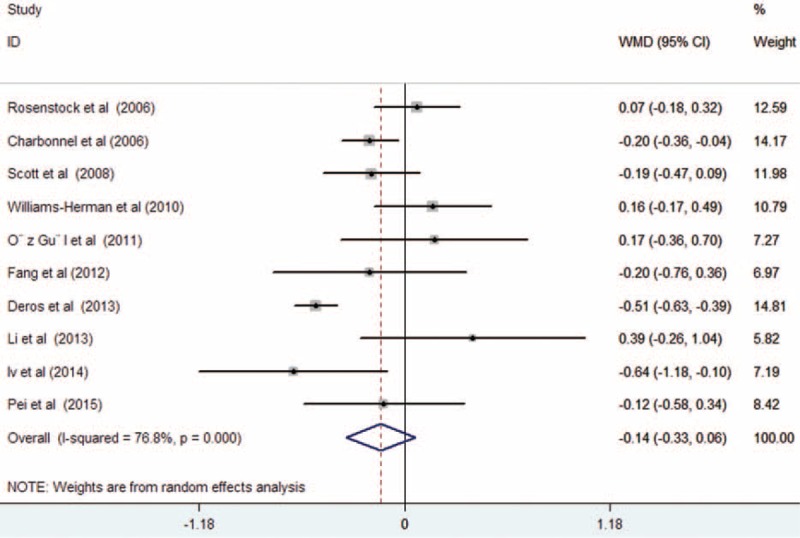
Forest plots showing weighted mean difference and 95% confidence interval for serum total cholesterol levels comparing sitagliptin alone or in combination with other antihyperglycemic agents to controls in a random effects model.

All the included trials reported the effect of treatment on serum TG levels. As shown in Figure 3, there was substantial heterogeneity among the included trials (*I*^2^ = 74.2%, *P* < 0.001); treatment with sitagliptin was associated with a significant decrease in serum TG levels (WMD −0.24 mmol/L; 95% CI −0.40 to −0.09; *P* = 0.002) compared with controls in a random effect model. Begg rank correlation test (*P* = 1.000) and Egger regression test (*P* = 0.106) did not reveal the evidence of publication bias. Ten trials^12–20,22^ reported the effect of treatment on serum HDL-C levels. As shown in Figure 4, there was no substantial heterogeneity across the 10 trials (*I*^2^ = 40.3%, *P* = 0.089), so we selected a fixed-effect model. Sitagliptin slightly increased serum HDL levels (WMD 0.05 mmol/L; 95% CI 0.02–0.07; *P* = 0.002) compared with the controls. Both Begg rank correlation test (*P* = 1.000) and Egger regression test (*P* = 0.164) for HDL-C did not suggest significant publication bias. However, there was no statistical significance in the comparison of serum levels of LDL-C (WMD −0.07 mmol/L; 95% CI −0.22 to 0.08; *P* = 0.337) with substantial heterogeneity (*I*^2^ = 75.8%, *P* < 0.001; Figure 5) and TC (WMD −0.14 mmol/L; 95% CI −0.33 to 0.06; *P* = 0.177) with obvious heterogeneity (*I*^2^ = 76.7%, *P* < 0.001; Figure 6). Egger tests for LDL-C and TC indicated significant publication bias in the meta-analyses (*P* = 0.047 and 0.069, respectively) but not in the Begg rank correlation test (*P* = 0.533 and 1.000, respectively).

### Subgroup Analysis and Sensitivity Analyses

Subgroup analysis (Table 2) was conducted by the intervention type (sitagliptin alone or sitagliptin in combination with other antihyperglycemic agents) and treatment duration (<18 or ≥18 weeks). The results indicated that sitagliptin alone achieved greater improvement in serum TG, TC, and HDL-C levels. Long-term treatment with the sitagliptin appeared to be associated with greater reduction in serum TG and HDL levels. Sensitivity analyses revealed that there was no change in the direction of the pooled WMD of lipid parameters when any one study was excluded (data not shown).

**TABLE 2:**
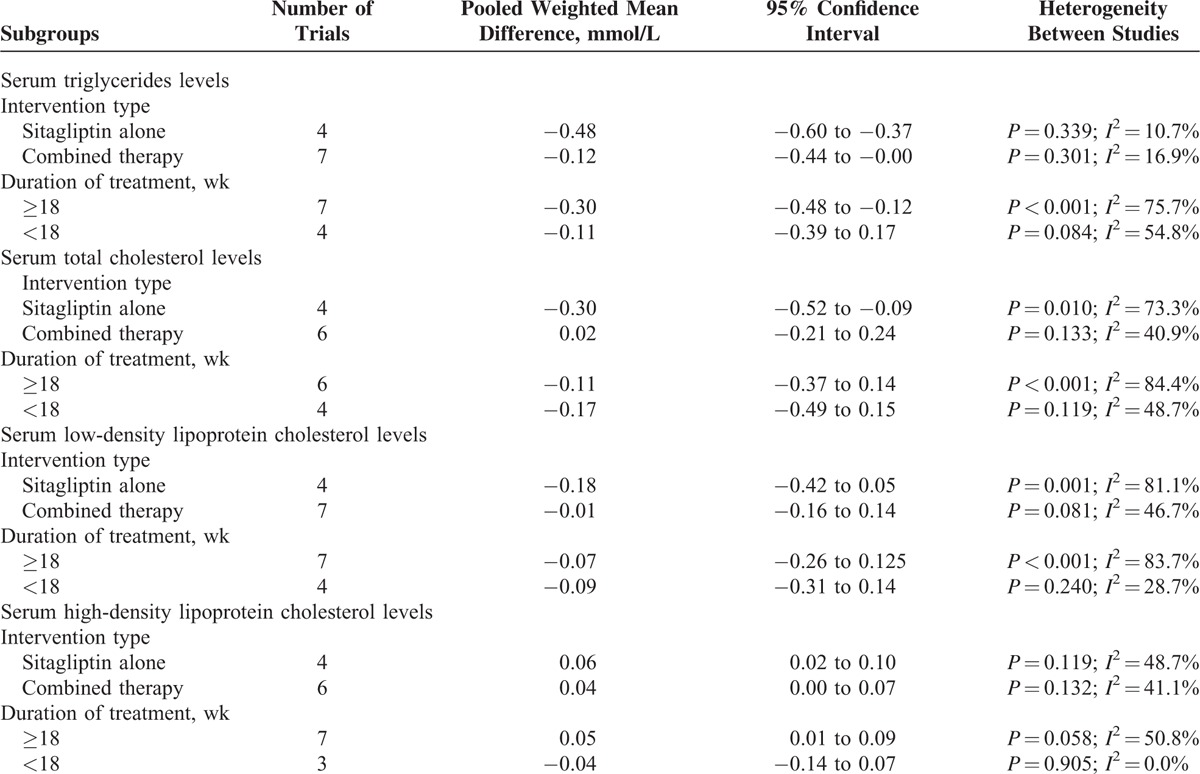
Subgroup Analyses of Serum Lipid Levels

## DISCUSSION

This is the first meta-analysis designed specially to assess the effects of sitagliptin on lipid profiles in patients with type 2 diabetes mellitus. The findings of this study suggest that sitagliptin alone or in combination significantly improved TG and HDL-C in patients with type 2 diabetes. Long-term treatment appeared to achieve better effects on serum TG and HDL-C levels. Sitagliptin alone achieves greater improvement in serum levels of TG, TC, and HDL-C than those in combination with other antihyperglycemic agents, which makes it a favorable therapeutic strategy for a person with diabetes along with dyslipidemia. However, the reductions in these variables are relatively small; whether the reduction is clinically relevant needs further investigation.

Low HDL-C level was the most frequent type of lipid abnormality in patients with type 2 diabetes.^23^ Current guideline on the treatment of blood cholesterol recommended the correction of low HDL-C and high TG levels among a person with type 2 diabetes.^24,25^ Single antihyperglycemic agent or in combination use could achieve target glycemic control in type 2 diabetic individuals. However, most of antihyperglycemic agents had a little impact on blood glucose levels. Therefore, diabetic patients with dyslipidemia represented a population with an excessive risk of cardiovascular disease.

DPP-4 inhibitors are a class of oral antihyperglycemic agents for treatment of type 2 diabetes.^4^ The effects of DPP-4 on lipid profiles in patients with type 2 diabetes have been widely investigated. Sitagliptin is a potent and well tolerable DPP-4 inhibitor. Our meta-analysis indicated that the overall effect of sitagliptin alone or in combination therapy on serum lipid improvement was approximately −0.24 mmol/L for TG and 0.14 mmol/L for HDL-C, respectively. Subgroup analyses showed that treatment with sitagliptin alone achieved greater improvement in serum TG, TC, and HDL-C levels than those in combination with other antihyperglycemic agents. Long-term treatment (≥18 weeks) with the sitagliptin appeared to achieve greater lipid-lowing effects than those with treatment less than 18 weeks. In addition, treatment with sitagliptin was associated with a significant decline in carotid intima-media thickness.^26,27^ Carotid intima-media thickness is a marker of subclinical atherosclerosis. Together, these findings revealed that sitagliptin had additional specific benefits on lowering TG, TC, and HDL-C levels, and provided potential for cardiovascular prevention.

Several studies not satisfying our inclusion criteria also investigated the effects of sitagliptin and incretin-based therapies on serum lipids. Shigematsu et al^28^ reported that sitagliptin significantly reduced serum TC, LDL-C, and non-HDL-C, particularly in patients with high baseline TG concentrations after 12 weeks’ treatment. Serum levels of TC and TG decreased after 3 months of treatment with sitagliptin in a prospective, open-label, multicenter trial.^29^ A retrospective study^30^ indicated that administration of sitagliptin significantly improved serum levels of TG, TC, and LDL-C except for serum HDL-C levels in patients with type 2 diabetes during 90 to 365 days follow-up period. Collectively, sitagliptin alone or in combination resulted in a more favorable lipid profile. However, the underlying mechanisms of sitagliptin in improving lipid are not fully elucidated. The beneficial effect of sitagliptin on serum lipid parameters could be partly explained by an improvement in glycemic control and insulin resistance, weight loss, or delayed gastric emptying. Incretin-based therapies also exhibited beneficial effects on lipoprotein metabolism.^31,32^ Apart from the effects on lipid parameters, sitagliptin and incretin-based therapies could improve oxidative stress and heme oxygenase-1, reduce markers of systemic inflammation, and improve endothelial dysfunction.^33,34^

Several limitations of this study should be considered. First, the primary outcome measures in most trials were glycemic control, and lipid determination may have been not very well-standardized. Second, substantial heterogeneities (*I*^2^ from 40.3% to 76.7%) existed in this meta-analysis, which may partly be explained by the patients’ characters, intervention type, and treatment duration. Third, potential publication bias cannot be excluded mainly due to the fact that lipid parameters were not the primary endpoint or not reported in most of the related studies. Moreover, evidence of publication bias was observed in Egger tests for pooled LDL-C and TC (*P* = 0.047 and 0.069, respectively). Finally, whether administration of sitagliptin achieved a greater reduction in cardiovascular events needs further investigation.

## CONCLUSIONS

This meta-analysis reveals that sitagliptin alone or added to other antihyperglycemic agents significantly improved serum TG and HDL-C levels in patients with type 2 diabetes mellitus. Sitagliptin alone achieves greater improvement in serum levels of TG, TC, and HDL-C. However, whether the use of sitagliptin can decrease cardiovascular events remains unclear.
